# Uncovering the Enigma of the C-shaped Root Canal Morphology

**DOI:** 10.7759/cureus.61883

**Published:** 2024-06-07

**Authors:** Ankita Burse, Joyeeta Mahapatra, Amit Reche, Srushti S Awghad

**Affiliations:** 1 Department of Conservative Dentistry and Endodontics, Sharad Pawar Dental College and Hospital, Wardha, IND; 2 Department of Public Health Dentistry, Sharad Pawar Dental College and Hospital, Wardha, IND

**Keywords:** hertwig’s epithelial root sheath, complex root canal morphology, backfill technique, c-shaped root canal configuration, c-shaped canal

## Abstract

A C-shaped canal describes an anatomical configuration of a tooth’s root canal that resembles the alphabet C when viewed occlusally in a prepared access cavity. In the second molar of the maxillary arch, the root canals unite into a single, continuous, extensive root canal morphology to form a C-shaped canal. The natural crevices found in tooth roots where blood vessels and nerves are housed are called root canals. The frequently referred etiology resulting in the development of the C-shaped canal arrangement is the inability of Hertwig’s epithelial root sheath to undergo fusion. The occurrence of the C-shaped canal anatomic variation varies among populations, with the majority of cases occurring in mandibular second molars. C-shaped canals pose several challenges in endodontic treatment such as in their diagnosis, biomechanical preparation, debridement, and obturation.

Nevertheless, the desired result can be achieved with relative ease if one has a solid grasp of the different root canal configurations and uses the relevant clinical expertise. Therefore, three-dimensional radiography is utilized to help identify and negotiate C-shaped canals by enabling three-dimensional reconstruction of the root canal system. Efficient C-shaped canal configuration treatment may be attained using hand-driven and rotary instruments assisted by sonic or ultrasonic hand-pieces. Four alternative gutta-percha filling methods are used in C-shaped canals: core-carrier, ultrasonic compaction, cold lateral compaction, and single cone with injectable gutta-percha. The core-carrier technique is the most efficient obturation technique in the C-shaped canal. Calcium silicate materials (CSMs) are also used for the obturation of C-shaped canals. The most frequently used CSMs are mineral trioxide aggregate and biodentine.

## Introduction

The C-shaped root canal (RC) was first explained by Keith and Knowles. They observed that the root separates lingually 7 mm beneath the coronal surface into mesial and distal fangs. Labially, the radicular portion of the tooth displayed no sign of division [1]. A C-shaped canal usually describes a certain anatomical configuration of a tooth’s RC. The natural crevices found in tooth roots where blood vessels and nerves are housed are called RCs [2]. The C-shaped canal resembles the letter C when viewed in cross-section. This arrangement is frequently observed in mandibular molars, especially the second molars. A continuous ribbon-like or C-shaped construction is used in a C-shaped canal compared to the more common oval or circular RCs [2]. C-shaped canals can make RC treatments difficult. They can be harder to clean, shape, and fill. Dental practitioners need to be aware of discrepancies in RC anatomy to ensure thorough treatment and successful outcomes in dental procedures. Proper identification and management of these variations are crucial in any clinical scenario.

The description of the C-shaped RC complex and its implications for treatment can be traced back to 1979 by Cooke and Cox [3]. The account written by Manning on the RC morphology of second molars of the lower arch that reported regarding the C-shaped RCs dates back to 1908 and 1911. The author analyzed the skeletal remains of Neanderthals, the ancestors of the Mongolian population that encompasses Asians [4]. While the RC(s) travel from the cervical one-third of the root to the apex, the C-shaped canal structure exhibits disparities in their number and location [5].

Although C-shaped canal variations can occur in any mandibular molar, their prevalence has been well documented in the maxillary molars [6]. The distinct RC morphology of the C-shaped RC may be caused while performing endodontic procedures. At the time of RC negotiation, debridement, and obturation, the clinician may encounter challenges due to this morphology. In two-dimensional X-rays, this kind of configuration is difficult to diagnose. Efficient treatment of the C-shaped canal configuration may be attained using both hand-driven and rotary instruments assisted by sonic or ultrasonic hand-pieces [6].

The etiology of complex maxillary second molar RC system variants is majorly due to the fusion of roots which results in fused and C-shaped canals [7]. These RC arrangements can be irregular and unpredictable which poses a therapeutic difficulty. The C-shaped RC may appear in two different ways: either as one ribbon-shaped RC that extends from the canal opening to the apical part of the root or it can show signs of many canals hiding beneath the orifice [7]. The extension of a C-shaped aperture in the pulpal chamber reaching the apex of the RC is hard to verify. If an irregularly shaped area inside a C-shaped canal is incompletely cleaned and filled, then there may be some remaining soft tissue debris and virulent products that might generate inflammation leading to severe pain and endodontic flare-up. The treatment objective of the present case was to undertake a thorough cleaning and shaping of the C-shaped canal without unnecessary removal of the healthy tooth structure while maintaining the anatomy and achieving a three-dimensional fluid-tight seal of the RC.

Etiology

Since the C-shaped canal anatomy was found, numerous explanations have been proposed for its origins. The definition of teeth has since altered, however, it was once thought that teeth with C-shaped radicular portions resembled taurodonts. The frequently occurring etiology resulting in the development of the C-shaped RC arrangement is the inability of Hertwig’s epithelial root sheath (HERS) to undergo fusion [8]. A lingual groove will form if the HERS cut out to merge with the buccal surface whereas a buccal groove may form if the lingual sheath cut out to merge. If the sheath is cut out to merge on the radicular portion buccally and lingually, a cone/prismatic-shaped root will emerge. If there is little space between the RCs, fusion is more likely to occur [9]. Before the discovery of racial predisposition, damage caused by radiation or chemical interferences was thought to be the causative agent for the irregular fusion of the HERS. However, according to the current concept, this type of anomaly has a genetic basis [10]. In these teeth, the RCs unite into one single, continuous, extremely large RC morphology that can resemble the letter C [11].

Prevalence

Mandibular second molars show the greatest occurrence of C-shaped RC structures, followed by mandibular first molars and other teeth [12]. These types of canals have a continuous ribbon-like or ovoid-shaped arrangement. There are regional and population-based disparities in the incidence of C-shaped canals. Studies have generally shown that compared to other populations, some ethnic Asian groups had greater prevalence rates [12,13]. Mandibular second molars have C-shaped RCs, which have been the subject of numerous studies [12,13]. The reported incidence varies depending on the target population and technique. It can be as high as 22%. It is crucial to remember that the predominance might change. The literature contains extensive documentation of the anatomic variety of canals [8]. The occurrence of the C-shaped RC anatomic variation fluctuates among populations, with the majority of cases occurring in mandibular second molars [12-14].

## Case presentation

A 57-year-old female patient came to the clinic with the chief complaint of pain in the upper left posterior region of the jaw for eight days. The pain started while eating food and subsided within two to three minutes of eliminating the stimuli. There was a history of nocturnal pain.

**Figure 1:**
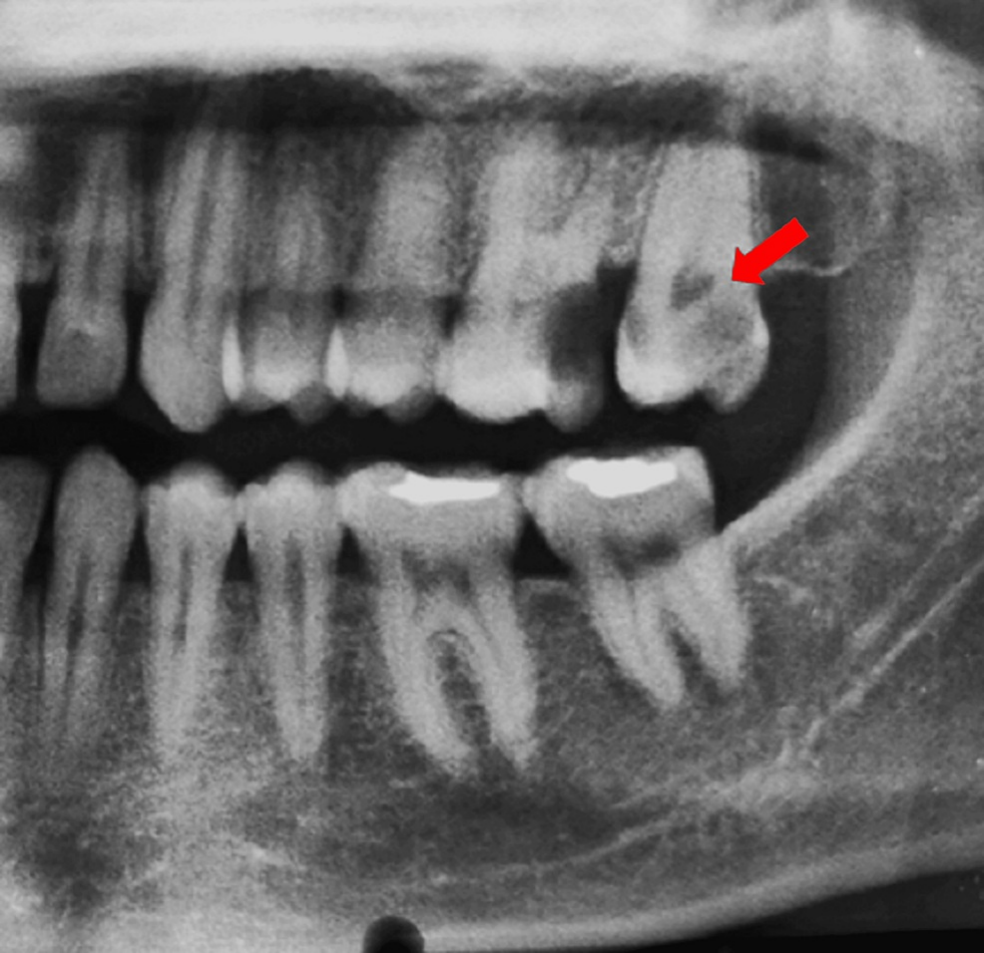
Preoperative radiographic view of the maxillary and mandibular left region of the jaw. The arrow showing the C-shaped canal with tooth number 27.

There was no relevant past medical and dental history. The face was bilaterally symmetrical. The temporomandibular joint was smooth and synchronous with no clicking sound. The lips were competent. On intraoral examination, all the teeth were present. Disto-occlusal carious involvement was seen with tooth number 26 which was also tender on vertical percussion. Occlusal caries was detected with tooth number 27 which was also tender on vertical percussion. Neural sensibility testing (cold test) of teeth numbers 26 and 27 showed delayed response compared to the contralateral control teeth with teeth numbers 16 and 17. Radiographic examination using intraoral periapical radiograph showed radiolucency involving enamel, dentin, and pulp in both teeth (26 and 27), which suggested the presence of dental caries. Widening of the periodontal ligament space was seen with teeth numbers 26 and 27, suggestive of apical periodontitis. An anatomical variation of root fusion was seen with tooth number 27 (Figure 1). Hence, the final diagnosis was symptomatic irreversible pulpitis with apical periodontitis with teeth numbers 26 and 27.

Local anesthesia was administered with 2% lignocaine hydrochloride with adrenaline (at a concentration of 1:2,00,000). This was followed by rubber dam isolation of teeth numbers 26 and 27. Access opening was done for both teeth using a round bur (BR-45, Mani, Japan) and a safe-end bur (EX-24, Mani, Japan). On the pulpal floor of tooth number 27, a C-shape was discovered. The configuration of the C-shaped canal was traced using a DG-16 Explorer (GDC Endo Explorer DG 16) which extended in the mesiodistal direction on the buccal half of the pulpal floor. A palatal canal was also seen in the same pulp chamber. Pulp extirpation was performed followed by rinsing of the cavity with saline. Patency filing was done up to the number 15 K-file in all canals. Working length was determined with the help of an electronic apex locator (J Morita Root Zx Mini, Tokyo, Japan). Both the buccal (C-shaped canal) and the palatal canal had a working length of 19 mm each with 27.

Tooth number 26 displayed a normal canal configuration, i.e., the presence of an oval-shaped configuration throughout the length of the RC. Conventional endodontic treatment was performed with tooth number 26.

**Figure 2:**
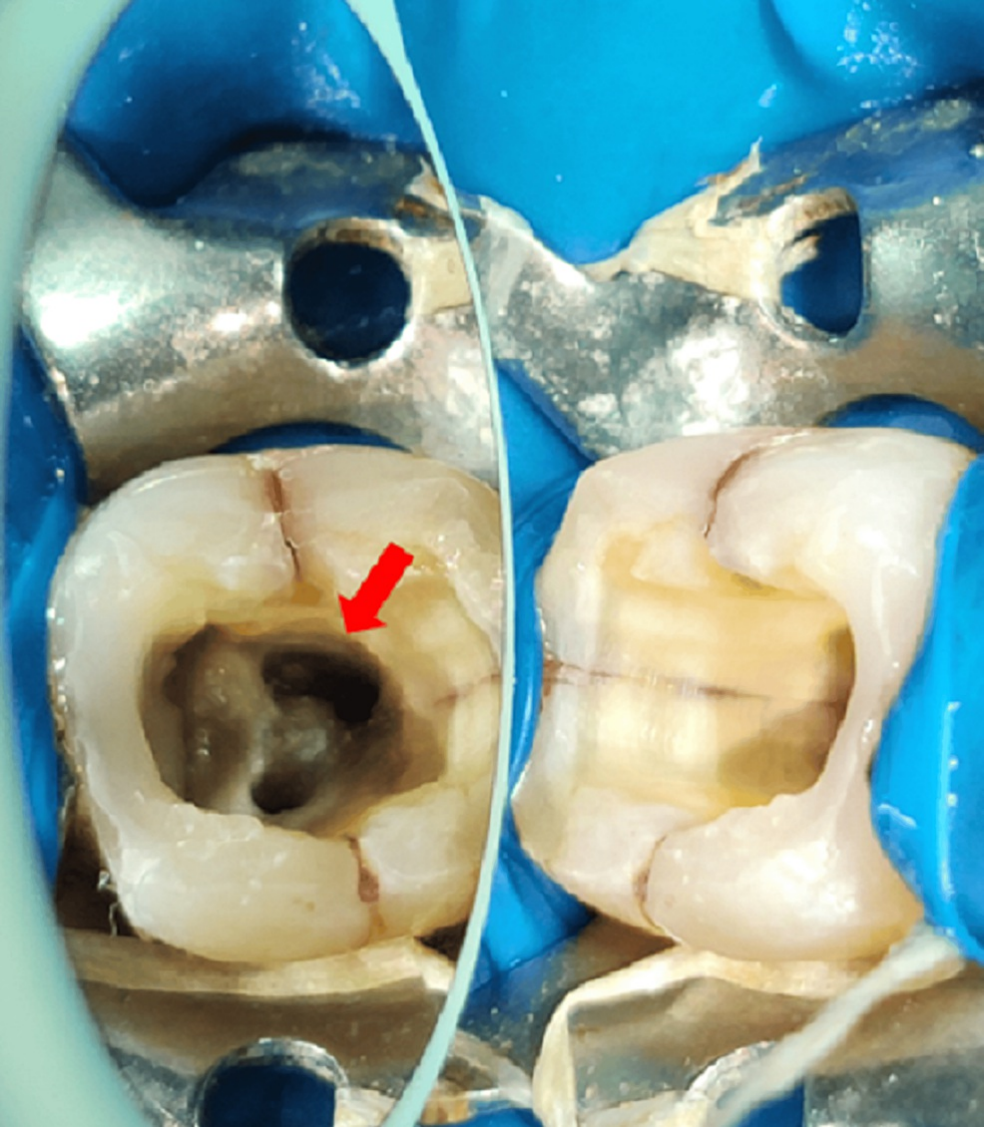
Clinical view of the access opening with tooth number 27. The arrow shows the C-shaped canal configuration with tooth number 27.

For the endodontic treatment of tooth number 27 which displayed a C-shaped canal configuration, biomechanical preparation was done using the crown down technique, followed by the sequential use of nickel-titanium ProTaper rotary file (Dentsply Maillefer, Bassllaigues, Switzerland). First, the SX, S1, and S2 shaping files were used sequentially. The canals were then prepared up to the finishing files F3 (Figure 2).

Circumferential filing was undertaken for the C-shaped canal. At every step of filing, irrigation was done alternatively with saline and 3% sodium hypochlorite (NaOCl). An inter-appointment dressing of calcium hydroxide was done for teeth numbers 26 and 27. The patient was recalled after one week for the evaluation of signs and symptoms related to the concerned teeth.

**Figure 3:**
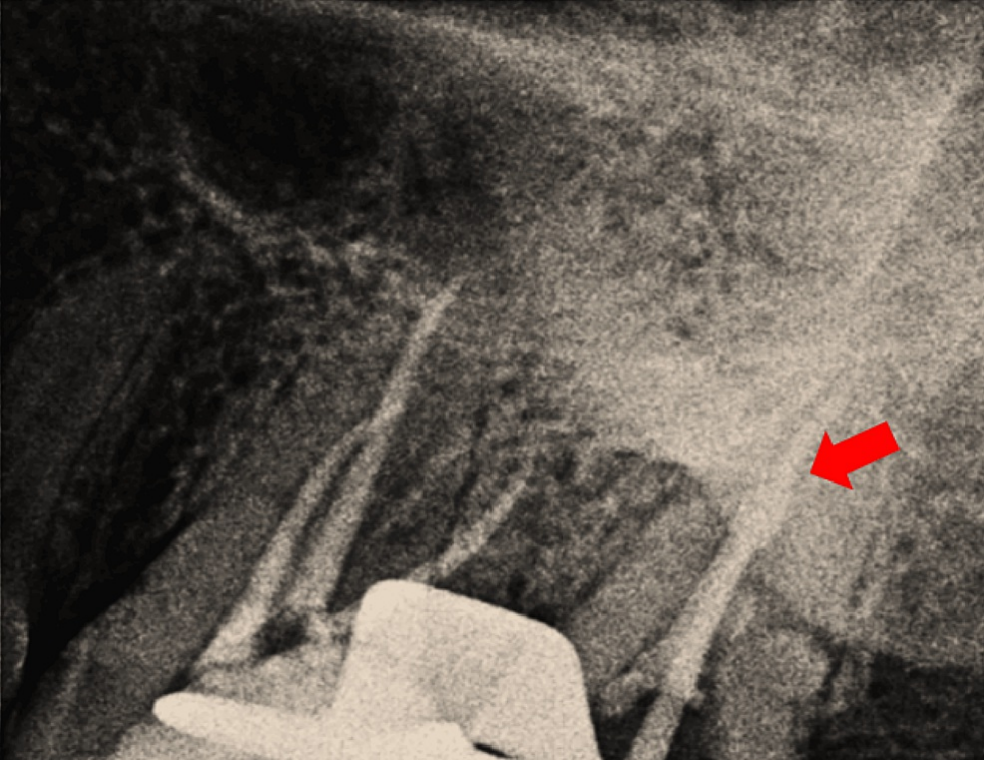
Intraoperative radiographic view of the maxillary left posterior region of the jaw. The arrow shows the overlapping master cones in both the buccal and palatal canal of tooth number 27.

Non-standardized gutta-percha (GP) corresponding to the dimension of the F3 ProTaper file was taken as the master apical cone for both the C-shaped buccal canal and the palatal canal of tooth number 27. The position of the master cone with the radiographic apex was verified with a periapical radiograph (Figure 3). After attaining the cone fit, the canals were again thoroughly irrigated with NaOCl and saline followed by drying with paper points. They were then coated with the sealer (Dia-Proseal, South Korea) with the help of their respective master cones. The palatal canal was obturated by the conventional single-cone obturation technique.

**Figure 4:**
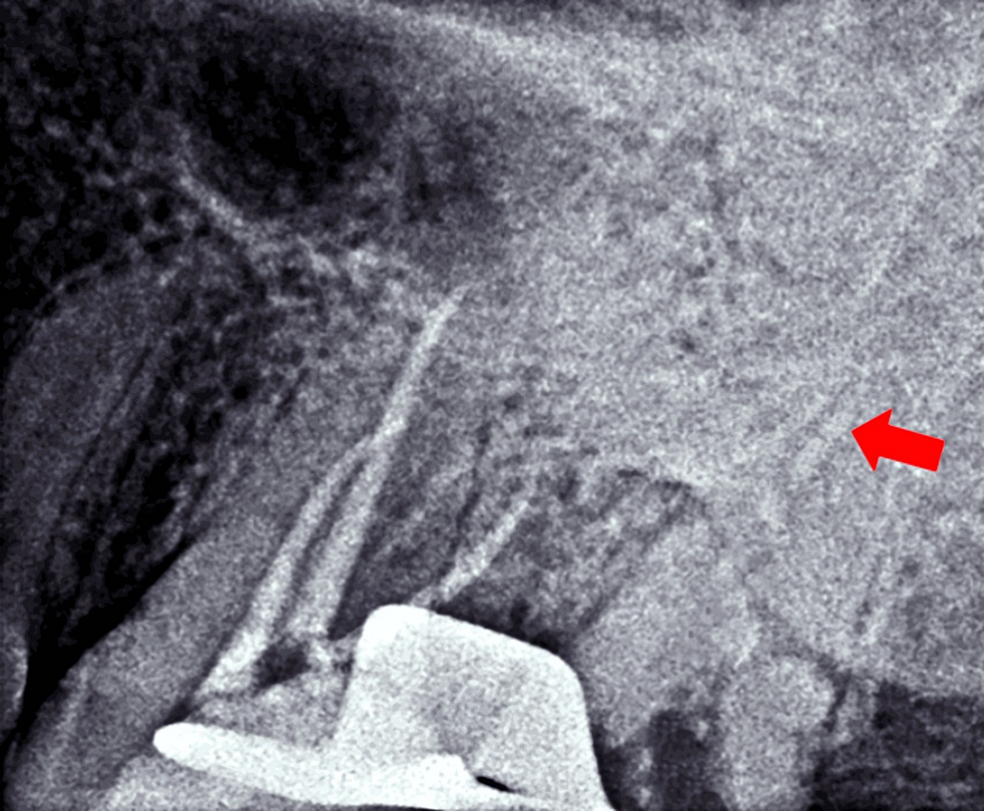
Intraoperative radiographic view of the maxillary left posterior region of the jaw. The arrow shows the 5 mm apical plug placed for the sectional obturation in tooth number 27.

The obturation of the C-shaped RC of tooth number 27 was done using the warm vertical compaction technique. A GP plug of 5 mm was established in the apical third of the RC. This was undertaken by first adjusting the length of the RC plugger to 14 mm length using a stopper and an Endogauge (Densply Mini Endo Bloc). The tip of the plugger was then heated to redness and inserted inside the C-shaped canal to achieve a GP plug of the desired length. The position and length of the GP plug were verified with a periapical radiograph (Figure 4).

**Figure 5:**
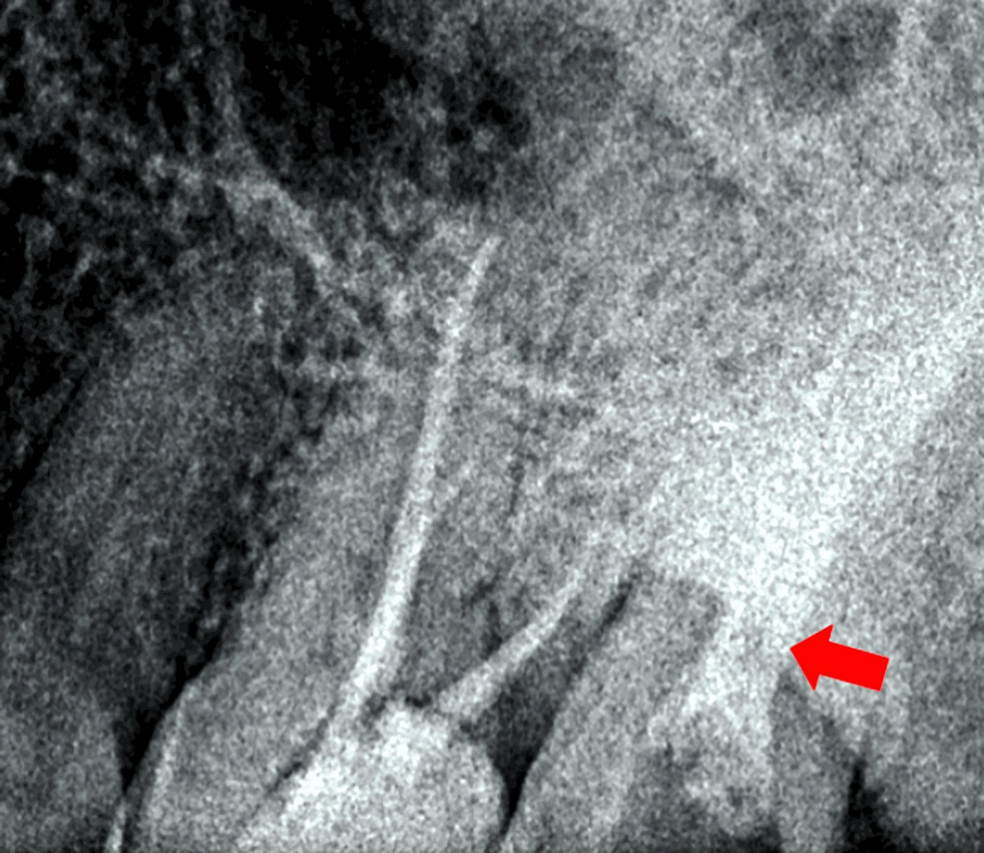
Radiographic view of the maxillary left posterior region of the jaw. The arrow shows the completed obturation in tooth number 27.

Then, the rest of the canal was filled with thermoplasticized GP using the thermoplastic backfill technique apico-coronally. After filling the canal, the plugger tip was again used to compact the GP. This was done until there was complete obturation up to the canal orifice (Figure 5).

**Figure 6:**
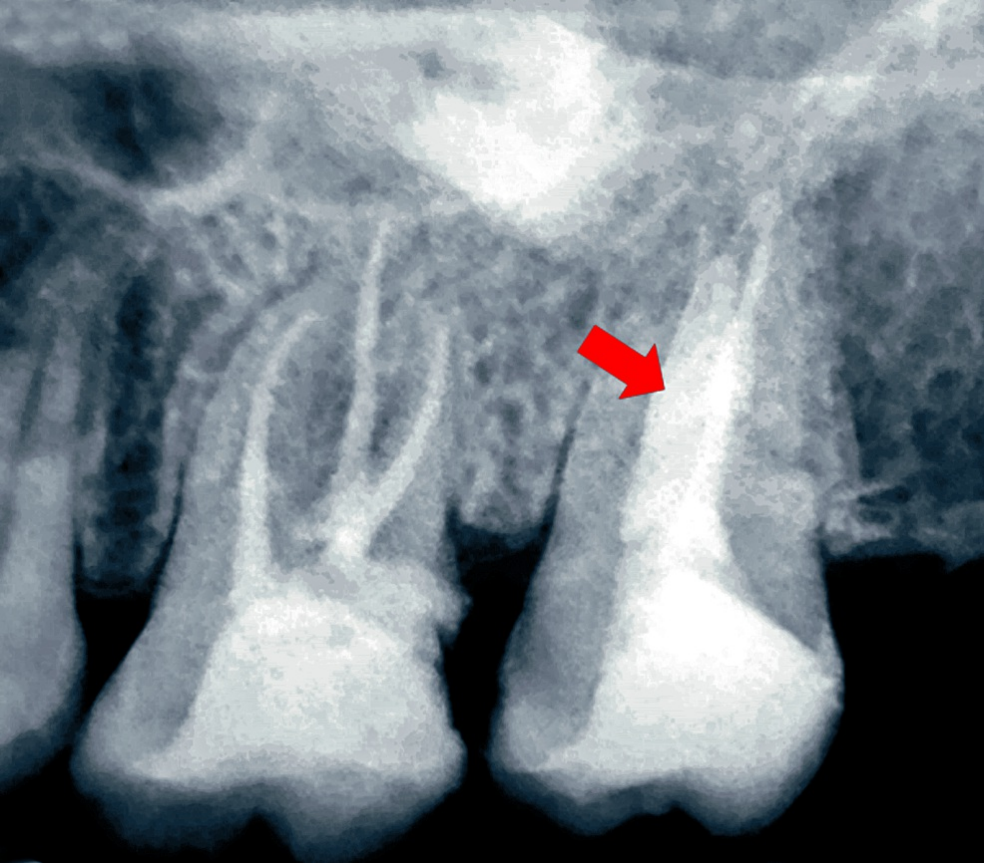
Follow-up radiographic view of the maxillary left posterior region of the jaw after one month. The arrow shows the obturated C-shaped canal of tooth number 27.

Post-endodontic restoration was done using resin composite in teeth numbers 26 and 27. The floor of the pulp was sealed with 2-3 mm of flowable composite (3M ESPE, Germany). The rest of the chamber was filled with packable resin composite (Spectrum Micro hybrid, Brazil). The patient was then given a crown prosthesis with 26 and 27. Radiographic follow-up was done at one month (Figure 6). During this recall period of one month, the patient did not show any clinically relevant signs or symptoms.

## Discussion

C-shaped canal shows equal gender predilection [6]. Fan et al. classified C-shaped canals into three types based on their radiographic appearance [14]. In type I, the C-shaped canal system gives the appearance of two distinct canals because of the presence of a thin isthmus connecting the mesial and distal canals that are not detected radiographically. In type II, the mesial and distal canals are two separate canals on the radiograph as they take different courses to the apex in the true sense. In type III, one main canal takes its course to the apical foramen giving the radiographic picture of a distinct canal while the other accessory canals proceed close to or within the fused area, that is, the “web” between the two main roots in the apical third [6].

The most complex RC systems are seen in maxillary molars in permanent dentition as they have more roots compared to the mandibular molars and other teeth [12,13]. Few studies have displayed that the maxillary second molars can have a C-shaped canal arrangement as these teeth often have three roots and three to four RC. A few studies have described the occurrence of RCs with this arrangement [13,15]. Globally, the upper second molar C-shaped canal occurrence rate is 3.8%; however, the prevalence of the C-shaped RC in the upper first molar was estimated to be 1.1% [16].

To ensure maximum tissue removal, circumferential filing should be used with caution. Calcified portions of the pulpal chamber must be cleared using ultrasonic tips to reveal the RC configuration fully [17,18]. The C-shaped canal’s intricate details need to be debrided by irrigation using 5.25% NaOCl. Sonics or ultrasonics should be used to activate the irrigation. The C-shaped canals are hard to obturate in three dimensions because of their complexity in anatomy. The thermoplasticized GP technique is always the treatment of choice for C-shaped canals [19]. Calcium hydroxide (Ca(OH)_2_) is the frequently used intracanal medicament to eliminate the infection of the RC system. Its high pH of 12.5 contributes to its antibacterial action [20,21]. Usually, it is used for seven to fourteen days. It should be eliminated before the final obturation as any remaining Ca(OH)2 in the RC may affect the sealer’s characteristics along with its sealing capacity [22]. Studies revealed that removing Ca(OH)_2_ from dentinal tubules of the RC walls, especially in the apical third of the canal, is very challenging [22,23].

Four alternative GP filling methods are used in C-shaped canals, i.e., core-carrier (ThermafilR), ultrasonic compaction, cold lateral compaction, and single cone with injectable GP (Obtura IITM, Malaysia and Indonesia). The core-carrier technique is the most efficient obturation technique in the C-shaped canal [24].

Calcium silicate materials (CSMs), the newest and most advanced materials, are undergoing extensive dentistry testing [25]. It has several advantageous uses in this industry, including pulp capping, root-end filling, mending broken tooth structure, obturation sealant, and pulpotomy. These cements have some desirable properties, such as good strength, radio-opacity, dimensional stability, antimicrobial activity, biocompatibility, bioactivity with the capacity to stimulate and modulate tissue formation, resistance to moisture sensitivity, non-absorbability to tissue fluid, and ease of manipulation. They also exhibit adequate sealing capacity by chemically bonding to the tooth structure [26,27]. Thus, they are frequently used for obturating a C-shaped canal. The most commonly used CSMs are mineral trioxide aggregate and biodentine. Of the two, biodentine comprises an enhanced calcium silicate-based material. It has excellent biocompatibility, improved mechanical properties, and excellent bioactive behavior and does not discolor the tooth [28-30].

## Conclusions

The cleaning, obturation, and restoration of the C-shaped RC seem to be challenging due to its variations in the anatomical features. For the successful treatment of a C-shaped RC, the clinician should have a thorough knowledge of these variations. Therefore, three-dimensional radiography is utilized to help with the correct diagnosis and negotiation of the C-shaped RCs by enabling three-dimensional reconstruction of the RC complex.
